# DHX9 interacts with APOBEC3B and attenuates the anti-HBV effect of APOBEC3B

**DOI:** 10.1080/22221751.2020.1725398

**Published:** 2020-02-14

**Authors:** Yanmeng Chen, Bocun Shen, Xiaochuan Zheng, Quanxin Long, Jie Xia, Yao Huang, Xuefei Cai, Deqiang Wang, Juan Chen, Ni Tang, Ailong Huang, Yuan Hu

**Affiliations:** Key Laboratory of Molecular Biology on Infectious Diseases (Ministry of Education), Institute for Viral Hepatitis, Department of Infectious Diseases, The Second Affiliated Hospital, Chongqing Medical University, Chongqing, People’s Republic of China

**Keywords:** Hepatitis B virus, APOBEC3B, DHX9, interaction, attenuate

## Abstract

Hepatitis B virus (HBV) is a partially double-stranded DNA virus that replicates by reverse transcription. We previously demonstrated that the host restriction factor-APOBEC3B (A3B) inhibited HBV replication which was dependent on its deaminase activity during reverse transcription. However, the host factors involved in the process of regulating the anti-HBV function of A3B are less known. In this research, to obtain a comprehensive understanding of the interaction networks of A3B, we conducted coimmunoprecipitation and mass spectrometry to identify A3B-interacting proteins in the presence of HBV. By this approach, we determined that DExD/H-box helicase 9 (DHX9) suppressed the anti-HBV effect of A3B, and this suppression was dependent on their interaction. Although DHX9 did not affect the deamination activity of A3B *in vitro* assay or the viral DNA editing of A3B in HepG2-NTCP cells that support HBV infection, it inhibited the binding of A3B with pgRNA. These data suggest that DHX9 can interact with A3B and attenuate the anti-HBV efficacy of A3B.

**Abbreviations:** 3D-PCR: differential DNA denaturation PCR; APOBEC3: apolipoprotein B mRNA-editing catalytic polypeptide 3; cccDNA: covalently closed circular DNA; co-IP: coimmunoprecipitation; DDX: DExD-box RNA helicases; HBc: HBV core protein; HBV: hepatitis B virus; HepAD38: HepG2 cell line stably transfected with HBV DNA; HepG2-NTCP: HepG2 cell line stably transfected with Na+/taurocholate cotransporter polypeptide; Huh7: human hepatoma cell line; pgRNA: pregenomic RNA; PPI: protein–protein interactions; RC DNA: relaxed circular DNA.

## Introduction

Hepatitis B virus (HBV) is a partially double-stranded DNA virus that specifically infects human hepatocytes [1]. HBV infection causes acute and chronic hepatitis [2]. Over 240 million people with chronic HBV carriers remain at a high risk of developing liver cirrhosis and hepatocellular carcinoma (HCC) [3, 4]. The mechanisms underlying the pathogenesis of HBV infection are still incompletely understood; however, the interactions between the virus and host factors play a vital role in determining virus-related diseases [5, 6].

HBV covalently closed circular DNA (cccDNA) is the template for the transcription of all four HBV mRNAs, including 3.5 kb RNA, preS/S mRNA, and X mRNA [7]. Subsequently, the pregenomic RNA (pgRNA) binds to HBV polymerase, triggering the polymerization of the core protein to form the nucleocapsid. In the nucleocapsid, reverse transcription takes place to synthesize the HBV genome [8].

In response to viral infection, host cells have evolved defense systems preventing virus invasion, one of which is “restriction factors”. Host restriction factors have been recently shown to block viral replication at specific steps of viral replication [9]. Meanwhile, the virus has also evolved sophisticated counteraction strategies such as protein degradation that antagonize restriction factors [10, 11]. Among these host restriction factors, the apolipoprotein B mRNA-editing enzyme catalytic polypeptide-like 3B (APOBEC3B, A3B) can be upregulated by lymphotoxin-β-receptor and edit HBV cccDNA in the nucleus, leading to cccDNA degradation in a cytidine deamination-dependent manner [12]. Our previous reports also indicated that A3B can inhibit viral replication during reverse transcription-dependent which is dependent on its deaminase activity [13], indicating that A3B exerts multifaceted antiviral effects against HBV. However, much less is known about how host factors are involved in this process. Thus, it is of great interest to identify host factors that are involved in the regulation of the anti-HBV function of A3B.

In this research, we performed immunoprecipitation (IP) coupled with mass spectrometry (MS) to systematically analyse A3B-interacting proteins in HEK293T cells in the presence and absence of HBV expression. Using these strategies, we determined that DExD/H-box helicase 9 (DHX9) interacts with A3B and attenuates the binding of A3B with HBV pgRNA, thus suppressing the anti-HBV effect of A3B. This research not only provides a full understanding of the host factors involved in the anti-HBV replication effect of A3B by MS analysis, but also provides a new sight into how DHX9 contributes to viral DNA replication partially by countering the anti-HBV function of A3B.

## Materials and methods

### Cell culture, siRNA and transfection

Human embryo kidney HEK293T cells, human hepatoma Huh7 cells, HepAD38 cells, and HepG2-NTCP cells were maintained as previously described [13]. HepAD38 cells were withdrawn from tetracycline treatment to induce HBV replication. Specific small interfering RNAs (siRNAs) targeting DHX9 or A3B and the control siRNA were synthesized by GenePharma Company (Shanghai, China) (Supplement Table 1). All cells were transfected with Lipofectamine 3000 (Cat. No. L3000-015, Invitrogen, California, USA) according to the manufacturer’s instructions.

### Plasmid constructs

The HBV expression plasmid was a vector carrying 1.1 copies of the HBV (genotype D) genome. The A3B expression plasmid with a 3× haemagglutinin (HA)-tag was constructed by Genecopoeia Company (Guangzhou, China). The full-length cDNA of human DHX9 cloned into the pCMV3 vector with a Flag-tag located in the N- terminus was constructed by Sino Biological Company (Beijing, China). For bacterial expression, the cDNA sequence of A3B was cloned in pGEX-6P-1 using *SaiI* and *NotI*. The different mutants of DHX9 were constructed by PCR-based mutagenesis. All primers are listed in Supplement Table 1.

### Immunoprecipitation and Western blotting

Total protein lysates of cells or liver tissues were obtained using RIPA lysis buffer (Cat. No. P0013B, Beyotime, Beijing, China) containing 1 mM PMSF and proteinase inhibitor cocktail (Cat. No. 04693159001, Roche, Mannheim, Germany). To detect the interaction of A3B with DHX9, coimmunoprecipitation (co-IP) was conducted as follows: after a 48 h transfection, HEK293T or Huh7 cells were lysed in RIPA buffer containing protease inhibitor cocktail (Roche) and 1 mM PMSF for 30 min on ice as described previously [13]. Cell lysates were clarified by centrifugation at 13,000 g for 5 min at 4°C, and then the supernatant was incubated with the antibody indicated in each figure overnight at 4°C. Immune complexes were pulled down with immobilized protein G agarose (16-266, Merck Company, Darmstadt, Germany) for 2 h at 4°C and then resolved by 10% SDS-PAGE. Western blotting was performed using the following antibodies: anti-HA (Cat. No. 26183, Thermo, Massachusetts, USA), anti-Flag (Cat. No. MA1-91878, Thermo, Massachusetts, USA), anti-A3B (Cat. No. bs-12494R, Bioss, Beijing, China), anti-DHX9 (sc-137232, Santa Cruz, USA), anti-GST (Cat. No. CW0084A, Cwbio, Beijing, China), anti-GFP (Cat. No. 13105-MM05, Sino Biological Inc. Beijing, China), anti-HBc (Cat. No. B0586, Dako, Glostrup, Denmark) and anti-GAPDH (Cat. No. AF0006, Beyotime, Beijing, China).

### RNA-protein immunoprecipitation

RNA-protein immunoprecipitation (RIP) was performed using a Magna RIP kit (Cat. No. 17-700, Millipore, MA, USA) according to the manufacturer’s instructions. Briefly, HEK293T or HepAD38 cells were transfected with the indicated plasmids, and 48 h later, the lysates were subjected to RIP. Aliquots (1/10 volume) of samples were detected by Western blotting for the desired proteins. Aliquots (1/10 volume) of samples were used for RNA extraction followed by real-time RT-PCR to measure HBV RNA levels in the input sample. The remaining samples were incubated with magnetic beads for immunoprecipitation with anti-HA antibody overnight. Aliquots (1/10 volume) of samples were used for Western blotting of immunoprecipitated proteins, and the remaining samples were subjected to proteinase K treatment and phenol/chloroform extraction. The relative expression of immunoprecipitated mRNAs was assessed by real-time RT-PCR. The primers that amplified three different regions of the HBV genome are listed in Supplement Table 1. HBV 3.5 kb RNA was detected by anchored PCR as previously reported [14].

### Virus production and HBV infection

For HBV inoculum production, supernatants of HepAD38 cells were precipitated with 10% PEG8000 overnight and concentrated. HepG2-NTCP cells were transfected with the plasmids or siRNA indicated in each figure and then inoculated with concentrated HBV viral particles at 1000 genome equivalents (GE) per cell in the presence of 4% PEG8000 for 16 h, as described previously [13].

### HBV DNA isolation and quantification of HBV DNA

HBV DNA from core particles in the cytoplasm was isolated as previously described [15, 16]. Briefly, the core-associated DNA was collected through a sucrose density gradient and purified by proteinase K digestion and phenol/chloroform extraction. The extracted DNA was separated in agarose gel and transferred onto a nylon membrane and detected using the DIG high prime DNA labelling and detection starter kit (Cat. No. 11585614910, Roche Diagnostics GmbH, Mannheim, Germany). Quantitative real-time PCR (qPCR) for measuring HBV core-associated DNA was conducted as previously described [17].

### 3D-PCR Analysis of HBV core-associated DNA

Differential DNA denaturation PCR (3D-PCR) was performed to detect G-A hypermutation induced by APOBEC3 proteins as described in our previous report [13].

### A3B deaminase activity assay *in vitro*

The deaminase activity of A3B was tested in a fluorescence-based deaminase activity assay with minor modification [18, 19]. Briefly, extracted proteins from cell lysates were incubated with 10 pmol of fluorescent oligonucleotides (FAM 5′-AAATTCTAATAGATAATGTGA-3 TAMRA′) and deaminase buffer containing 1 mM MgCl_2_, 100 mM Tris–Cl (pH 8.0), 500 mM NaCl, and 10 mM DTT in 96-well plates at 37°C for 2 h and treated with 1 μL of uracil DNA glycosylase (Cat. No. M0280S, New England Biolabs, USA), 200 mM Tris–HCl (pH 8.2), 10 mM EDTA, and 100 mM NaCl at 37°C for 45 min. After 0.6N NaOH was added to break the edited oligonucleotides, the released fluorescence was collected and measured on a Synergy plate reader (BioTek, VT, USA).

### GST pulldown *in vitro* assay

Expression of the recombinant A3B protein was conducted as follows. Briefly, *E. coli* BL21 cells were transformed with a pGEX-6P-1 plasmid containing the A3B cDNA sequence, and expression of the GST-A3B fusion protein was induced at 37°C with 0.5 mM IPTG. The bacterial cells were lysed by sonication and stored at −80°C for the GST pulldown experiment. Recombinant GST-A3B was incubated with cell lysates derived from HEK293T cells transfected with DHX9 expression plasmids and Glutathione Sepharose 4B (Cat. No. 17-0756-01, GE Healthcare, Sweden) on a rotator at 4 °C overnight. The beads were extensively washed with PBS buffer containing 1% Triton X-100, resuspended in SDS-PAGE loading buffer, boiled and then subjected to Western blotting.

### Immunofluorescence

The methods for measuring the cellular location of DHX9 truncated constructs by immunofluorescence have been described previously [20]. Briefly, Huh7 cells transfected with the indicated plasmids were fixed with 4% paraformaldehyde, followed by permeabilization with 0.5% Triton X-100 and blocking with 10% bovine serum albumin. The cells were incubated with the anti-Flag antibody and the Alexa Fluor 488-conjugated secondary antibody. Finally, the cells were treated with 4′,6-diamidino-2-phenylindole (DAPI), and visualized by a Leica confocal laser microscope.

### Mass spectrometry analysis and protein–protein interactions network construction

HEK293T cells were transfected with HA-A3B with or without HBV expression plasmids. After 48 h, the cell lysates were harvested and immunoprecipitated with an anti-HA monoclonal antibody. Then the immunoprecipitated proteins were separated by SDS-PAGE and stained with Coomassie brilliant blue. After the bands were excised from the gel lanes and digested, the potential proteins interacting with A3B were analysed by LC-MS (PTM Biolabs, Hangzhou, China) (Supplement Table 2).

To identify the involved functions of host proteins interacting with A3B with or without HBV, protein–protein interaction (PPI) networks were constructed with the STRING database, and GO annotation enrichment was performed.

### Statistical analysis

The statistical analyses were performed by GraphPad Prism 5. The statistical relevance of the differences between the two groups was tested by *t*-test. For groups of three or more, one-way ANOVA with a Tukey’s post hoc test was employed for analysis of variance. *p* < 0.05 was considered statistically significant.

## Results

### Identification of cellular proteins interacting with A3B and construction of the PPI network

**Figure 1.**
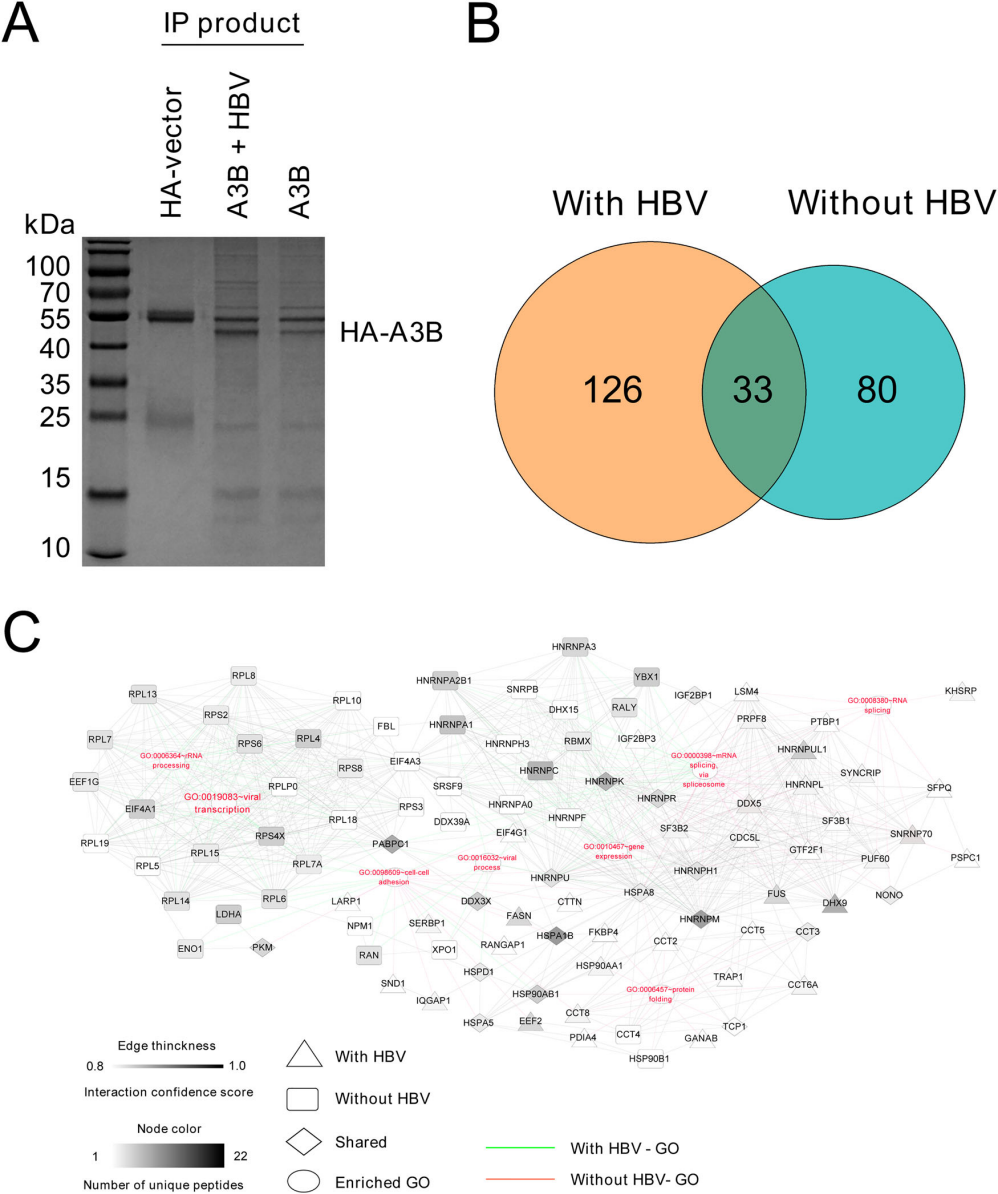
Identification and comparison of cellular proteins with or without HBV identified by IP-MS. (A) HEK293T cells were transfected with HA-tagged A3B with or without HBV replication plasmid. The cell lysates were coimmunoprecipitated using anti-HA antibody and analysed by SDS-PAGE, and then the gel was processed for MS. (B) Diagrams describe the number of proteins interacting with A3B identified in the MS data in the presence or absence of HBV. (C) A3B PPI constructed based on enriched GO annotations. Edge thickness describes the confidence score for interactions determined by the STRING database, and the node colour describes the number of unique peptides derived from the mass spectrometry analysis.

A3B is a nucleocytoplasmic shuttling protein, and it not only edits cccDNA in the nucleus [12] but also edits HBV DNA during reverse transcription in the cytoplasm [13]. In this regard, we sought to identify the possible targets located in either the cytoplasm or nucleus that can interact and potentially regulate the antiviral function of A3B in the presence of HBV replication. HA-tagged A3B was cotransfected with or without an HBV replication plasmid in HEK293T cells and immunoprecipitated using an antibody directed against HA. The co-IP products were subjected to SDS-PAGE and stained by Coomassie brilliant blue to confirm successful IP and then analysed by LC-MS. After LC-MS analysis, we identified 113 proteins that potentially interact with A3B in the absence of HBV, and 126 proteins exclusively interact with A3B in the presence of HBV (Figure 1(A,B)). To establish a comprehensive understanding of the involved function and interaction networks of host proteins interacting with A3B, we next conducted enrichment analysis based on Gene Ontology (GO) annotations. These annotations revealed that the HA-A3B interacting proteins function in specific mRNA metabolism, protein folding and viral process (Figure 1(C)). Among the candidates for A3B-interacting proteins, DHX9 was selected for further study, as we previously demonstrated that HBx induced upregulation of DHX9 by repressing its proteasome-dependent degradation, and DHX9 contributed to HBV DNA replication [20]; however, the underlying mechanism is unclear. Given that DHX9 interacts with A3B as demonstrated by MS data, we asked whether DHX9 promotes viral DNA replication by interacting with and attenuating the anti-HBV effect of A3B.

### DHX9 interacts with A3B

**Figure 2.**
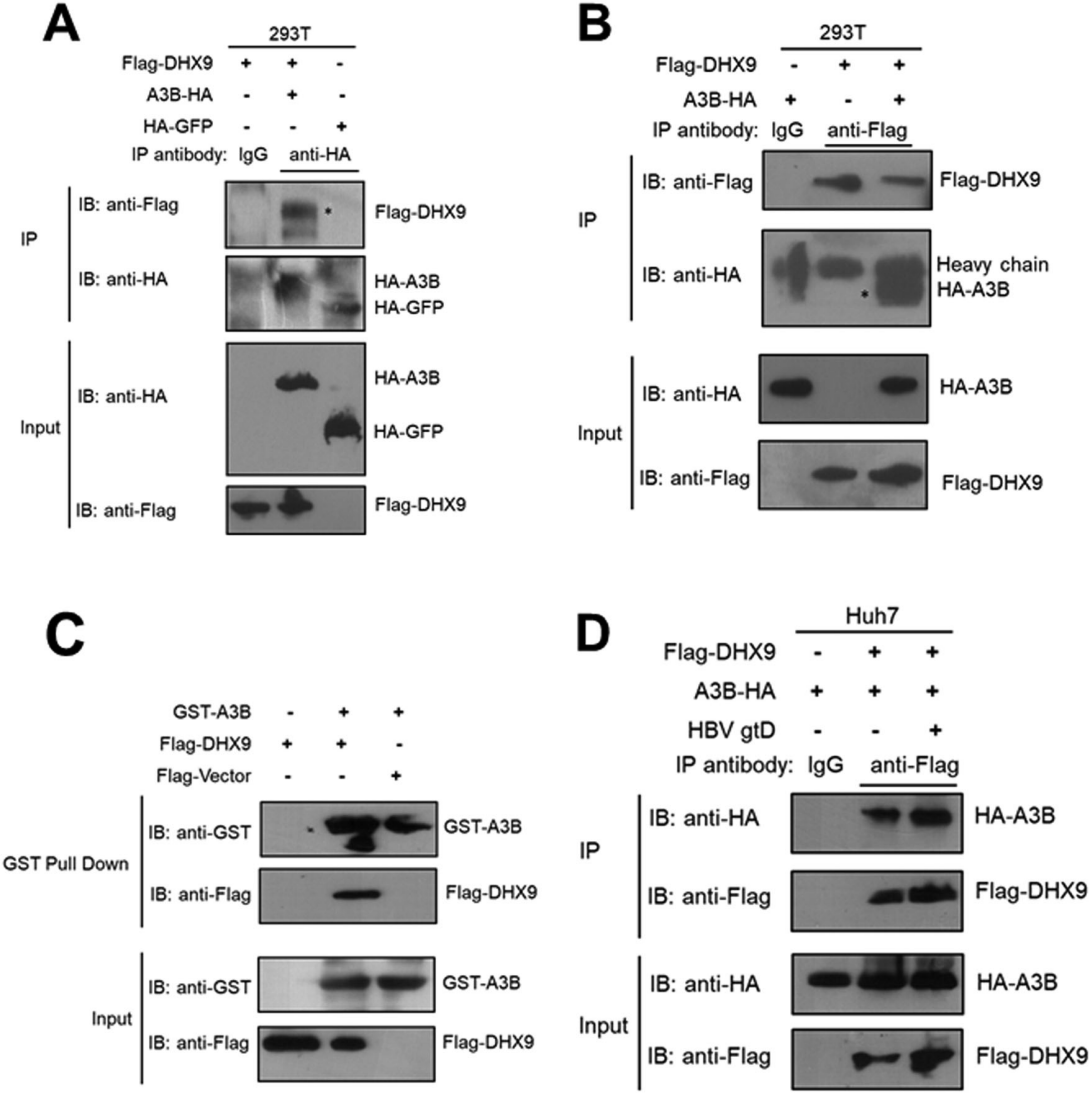
DHX9 interacts with A3B directly. (A) HEK293T cells were transfected with the HA-A3B expression plasmid alone or together with a Flag-DHX9 expression plasmid, and then the cell lysates were immunoprecipitated with control IgG antibody or anti-HA antibody and blotted with anti-HA antibody or anti-Flag antibody. (B) Reverse co-IP analysis of the interaction between A3B and DHX9 was performed by immunoprecipitation with anti-Flag antibody. (C) HEK293T cells were transfected with the Flag-DHX9 expression plasmid. After 24 h, the cell lysates were incubated with GST-A3B protein, and GST pulldown assays were conducted. The proteins bound to Glutathione Sepharose beads were blotted with the indicated antibodies. (D) The interaction of A3B and DHX9 was increased in the presence of HBV. Huh7 cells were cotransfected with HA-A3B and Flag-DHX9 expression plasmids in the absence or presence of the HBV replication plasmid (gtD), then co-IP assay and Western blotting were conducted as (B).

First, we validated whether DHX9 is physically associated with A3B by co-IP assay. Briefly, HEK293T cells cotransfected with HA-tagged A3B and Flag-tagged DHX9 plasmids were lysed and immunoprecipitated with anti-HA antibody, and then the IP products were analysed by Western blotting with an antibody specific to detect DHX9. As shown in Figure 2(A), Flag-tagged DHX9 could interact with HA-tagged A3B but not HA-tagged GFP in HEK293T cells, indicating that DHX9 specifically interacts with A3B. A reverse co-IP assay further confirmed that DHX9 was detected in A3B immunoprecipitates (Figure 2(B)). Moreover, we performed *in vitro* GST pulldown experiments. As shown in Figure 2(C), DHX9 interacted directly with A3B compared with the negative control. Moreover, we found that the levels of DHX9 bound to A3B were significantly enhanced in the presence of HBV (Figure 2(D)), which may be due to HBV infection-induced upregulation of DHX9. These findings prompted us to investigate the potential function of DHX9 in regulating the anti-HBV effect of A3B.

### DHX9 negatively regulates the anti-HBV replication effect of A3B

**Figure 3.**
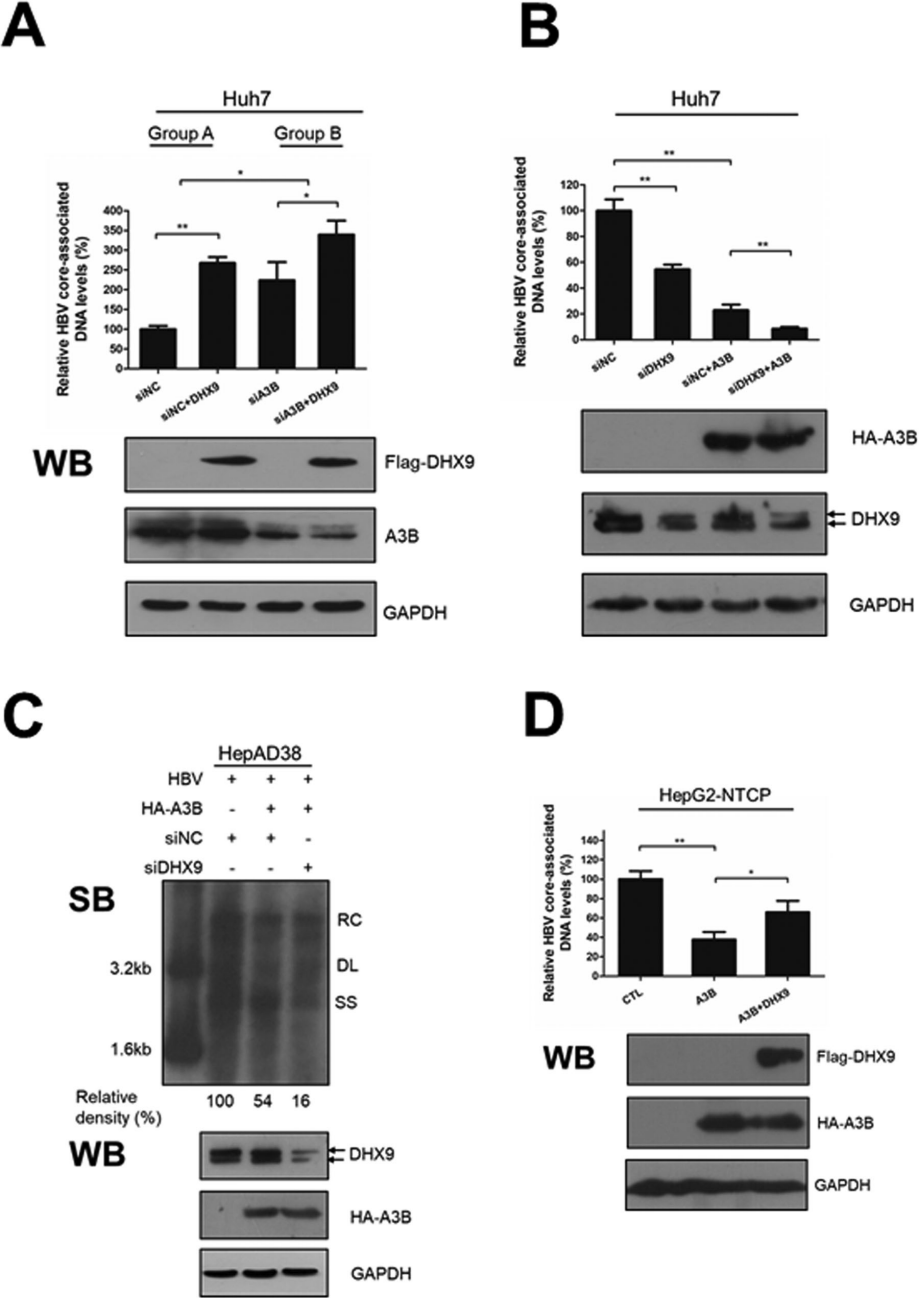
DHX9 attenuates the anti-HBV function of A3B in loss- and gain-of function experiments. (A) Huh7 cells were transfected with siRNA targeting A3B and then cotransfected with Flag-DHX9 and the HBV expression plasmid. Viral DNA was measured by qPCR. (B) Huh7 cells were first transfected with siRNA targeting DHX9 or control siRNA first and then cotransfected with HA-A3B expressing plasmid and HBV expression plasmid (ratio = 3:1). After 3 days, HBV DNA levels in cytoplasmic fractions were measured by qPCR, and the expression of the DHX9 and A3B proteins was analysed by Western blotting. (C) The intracellular HBV DNA in HepAD38 cells was extracted and measured by Southern blot. (D) HepG2-NTCP cells were transfected with HA-A3B alone or together with the Flag-DHX9 plasmid, followed by infection with HBV particles for four days. The viral DNA levels were measured by qPCR. RC: relaxed circular; DL: double-stranded linear; SS: single stranded. The mean ± SD of three experiments is represented. Statistical significance was determined by one-way ANOVA with Tukey’s post hoc test (*p < 0.05, **p < 0.01, ***p < 0.001).

Next, to determine whether DHX9 was involved in regulating the anti-HBV function of A3B, Huh7 cells were transfected with A3B-specific siRNA to knock down the expression of A3B in the presence or absence of DHX9, and then the HBV core-associated DNA levels were measured by qPCR. Overexpression of DHX9 led to a 2.7-fold increase in viral DNA level (Figure 3(A), group A), while in the A3B knockdown group, overexpression of DHX9 only led to a 1.5-fold increase in viral DNA levels, although it did not completely disappear (Figure 3(A), group B). This indicated that the contribution of viral DNA replication by DHX9 was to some degree decreased in the absence of A3B. Moreover, overexpression of A3B significantly reduced HBV DNA levels, while silencing the expression of DHX9 significantly promoted the anti-HBV effect of A3B (Figure 3(B)), indicated that DHX9 suppressed the anti-HBV effect of A3B. A Similar effect was also observed in HepAD38 cells (Figure 3(C)). Conversely, overexpression of DHX9 led to the significant attenuation of the anti-HBV function of A3B in HepG2-NTCP cells that support HBV infection (Figure 3(D)). Taken together, these data indicate that DHX9 contributes to viral DNA replication partially by attenuating the anti-HBV effect of A3B.

### DHX9 restricts the anti-HBV function of A3B dependent on its interaction with A3B

**Figure 4.**
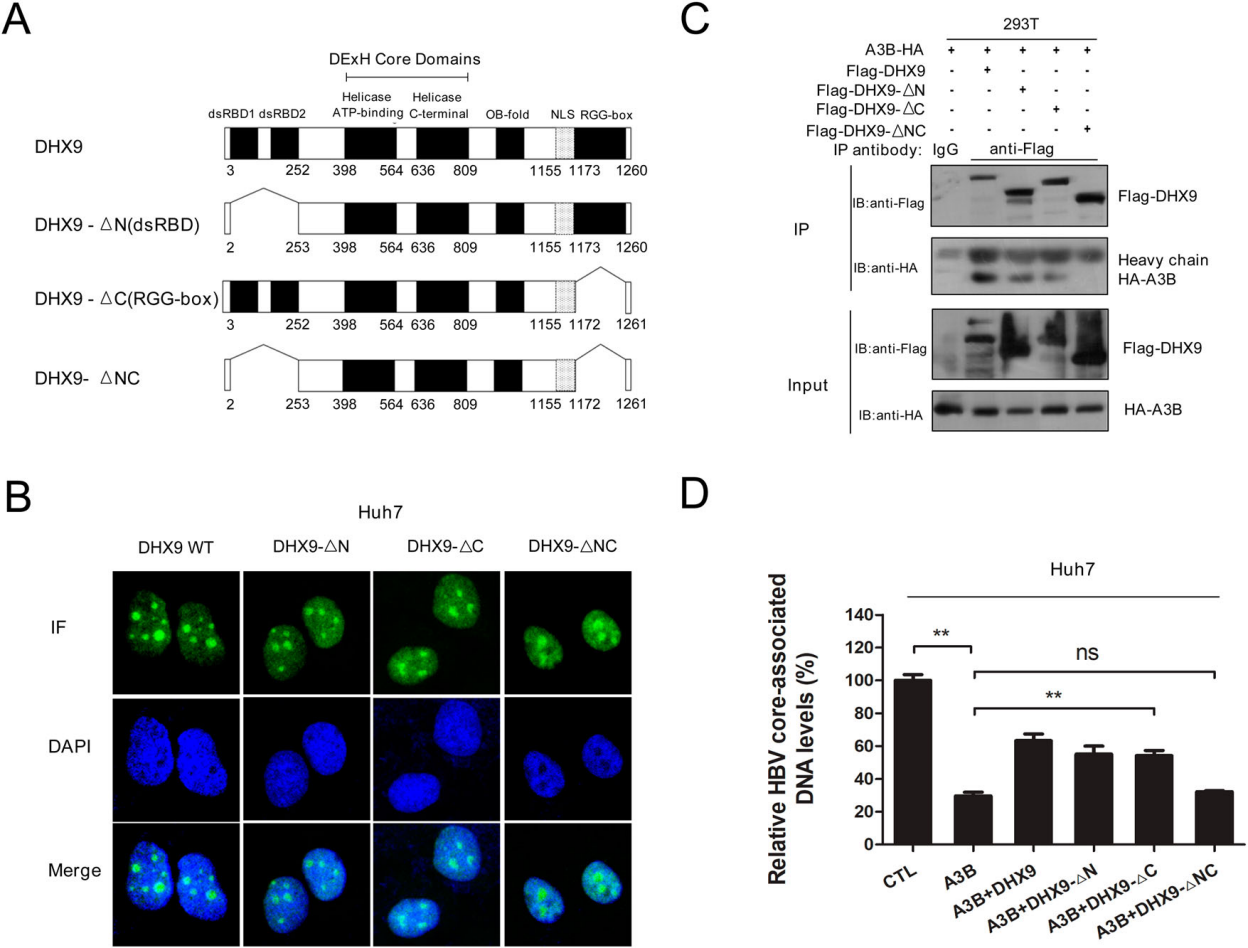
DHX9 suppresses the anti-HBV function of A3B in a manner dependent on their interaction. (A) Schematic diagrams of full-length DHX9 and truncation mutants. dsRBD: double-stranded RNA binding domains; OB fold: oligonucleotide/oligosaccharide binding fold; NLS, nuclear localization signal; RGG, arginine-glycine-glycine; (B) Analysis of the localization of DHX9 wild-type (WT) and truncation mutants in Huh7 cells by immunofluorescence. (C) Mapping of the DHX9 domain required for the DHX9/A3B interaction. Lysates harvested from HEK293T cells cotransfected with HA-A3B and Flag-DHX9 wild-type (WT) or truncation mutants were used for co-IP. (F) DHX9 attenuates the anti-HBV function of A3B in a DHX9/A3B interaction-dependent manner. Huh7 cells were cotransfected with HA-A3B and Flag-DHX9 WT or truncation mutants, and viral DNA levels were measured by qPCR. Statistical significance was determined by one-way ANOVA with Tukey’s post hoc test (*p < 0.05, **p < 0.01, ***p < 0.001, ns, not significant.).

Next, we explored the underlying mechanism by which DHX9 inhibits the antiviral activity of A3B. Given that DHX9 interacts with A3B, as demonstrated in Figure 2, we asked whether DHX9 inhibits the antiviral activity of A3B in a manner dependent on their interaction. DHX9 contains 2 double-stranded RNA binding domains (dsRBDs) at N-terminus, a helicase core domain at the centre and a glycine-rich RGG-box at the C-terminus (Figure 4(A)). To determine the domain required for DHX9–A3B interaction, we constructed serial deletion mutants of DHX9 (Figure 4(A)) and conducted immunofluorescence analysis. Considering that DHX9 lost the ability to stimulate HBV DNA replication when the nuclear import localization signal (NLS) of DHX9 was deleted in our previous report [20], the NLS region was not removed in these deletion mutants of DHX9. Immunofluorescence analysis confirmed that both the wild type (DHX9 WT) and mutants (DHX9-ΔN, DHX9-ΔC and DHX9-ΔNC) still predominantly localized to the nucleus in Huh7 cells. Then, these truncation constructs were used in co-IP analysis. As shown in Figure 4(C), HA-tagged A3B was pulled down by the DHX9, DHX9-ΔN and DHX9-ΔC proteins but not the DHX9-ΔNC deletion mutant protein. This indicated both dsRBDs and the RGG region of DHX9 served as the main binding region for A3B. Next, these truncation constructs of DHX9 were cotransfected with the HBV replication plasmid and HA-tagged A3B plasmid in Huh7 cells, and we observed that the attenuation in the presence of DHX9 of restriction of A3B disappeared in the DHX9-ΔNC deletion group but not in the other three groups (Figure 4(D)). This indicated that DHX9 inhibition of the anti-HBV function of A3B is dependent on their interaction.

### DHX9 does not show a significant effect on the in vitro deaminase activity or editing of HBV DNA by A3B

**Figure 5.**
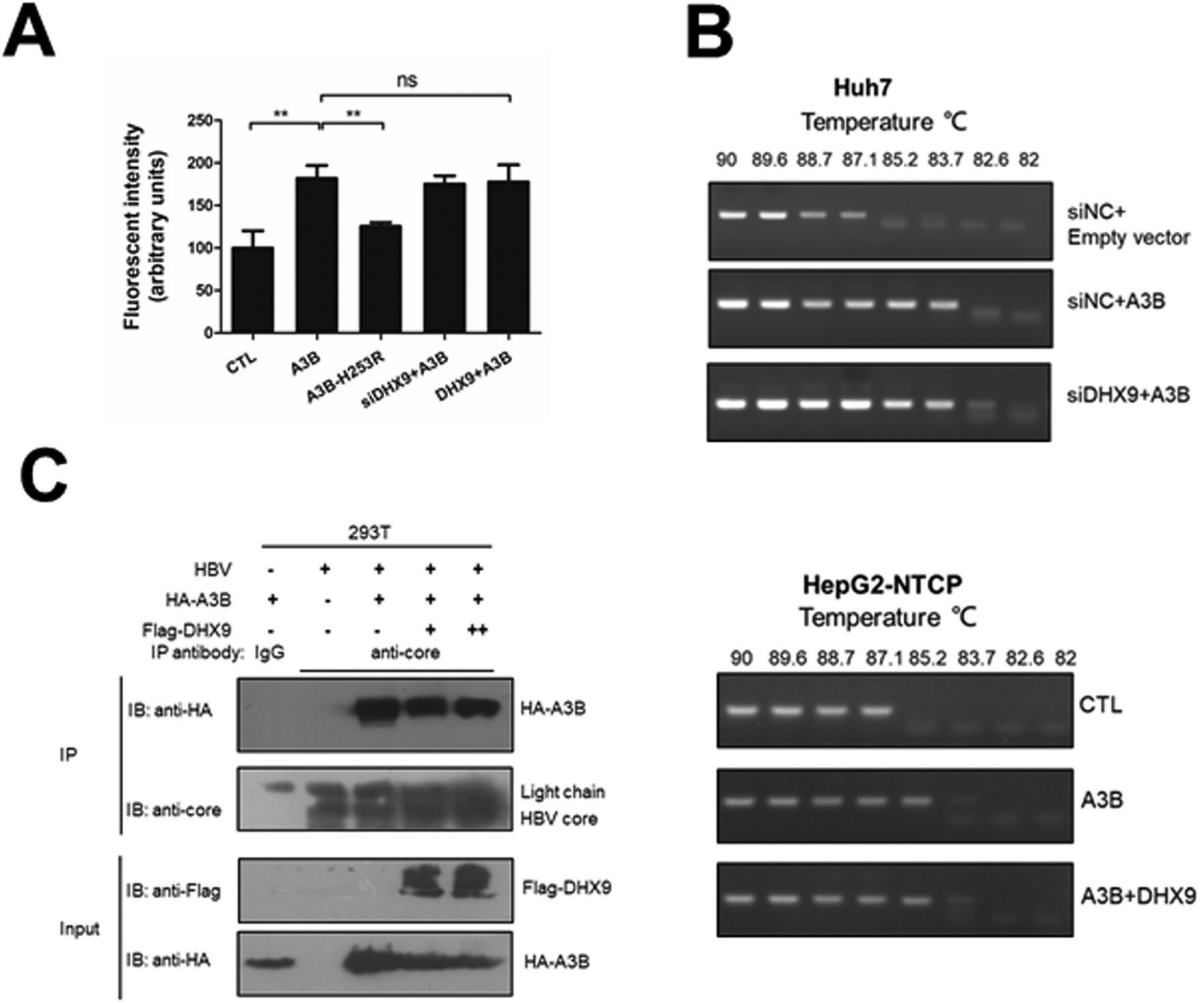
DHX9 does not regulate the *in vitro* deaminase activity or editing of HBV DNA by A3B. (A) Silencing or overexpression of DHX9 does not stimulate the deaminase activity of A3B. Huh7 cells were transfected with siRNA targeting DHX9 and then cotransfected with HA-A3B alone or together with Flag-DHX9 plasmid as indicated above. The cell lysates were used in a deaminase activity assay *in vitro* as described in the Materials and Methods. (B) HBV core-associated DNA from Huh7 or HepG2-NTCP cells cotransfected with the indicated plasmids was divided into aliquots: one for qPCR analysis in Figure 3(A) and another for 3D-PCR. (C) DHX9 does not affect the interaction between A3B and HBc. HEK293T cells were cotransfected with the indicated plasmids, and then the lysates were collected and subjected to co-IP assays using anti-HBV core antibody or mouse IgG.

As the A3B inhibition of HBV replication is dependent on its cytidine deaminase [13], we first tested whether DHX9 can affect the deaminase activity of A3B using a fluorescence-based assay *in vitro*. A3B can deaminate cytidine to uracil in a single-stranded DNA substrate. In the presence of uracil DNA glycosylase (UNG), uracil excision creates a break in the oligonucleotide, and the cleaved products with a fluorescent label can be measured on a fluorescence scanner [19]. We observed that the deaminase activity of A3B increased in the cell lysates of the A3B wild-type expression group but not in those of the deaminase-deficient A3B-H253R mutant. Knockdown of the endogenous expression or ectopic expression of DHX9 had no significant effect on the deaminase activity of A3B (Figure 5(A)). Moreover, we employed a 3D-PCR assay to test whether DHX9 affects the degree of editing viral DNA by A3B in HBV transfection-replication and HepG2-NTCP infection models. The HBV core-associated DNA in the presence of A3B was heavily edited; however, silencing or ectopic expression of DHX9 had no effect on the A3B editing of viral DNA (Figure 5(B)). Second, as binding with the HBV core protein is necessary for the A3B restriction of HBV DNA replication, we asked whether the binding of DHX9 with A3B attenuates the interaction of A3B and HBc. As shown in Figure 5(C), A3B could interact with HBc, which was in accordance with our previous report [13]. However, ectopic expression of DHX9 did not affect the interaction of A3B and HBc. Therefore, DHX9 restricts the anti-HBV function of A3B independent of affecting the deaminase activity of A3B or the A3B-HBc interaction.

### DHX9 attenuates the binding between A3B and HBV 3.5 kb RNA

**Figure 6.**
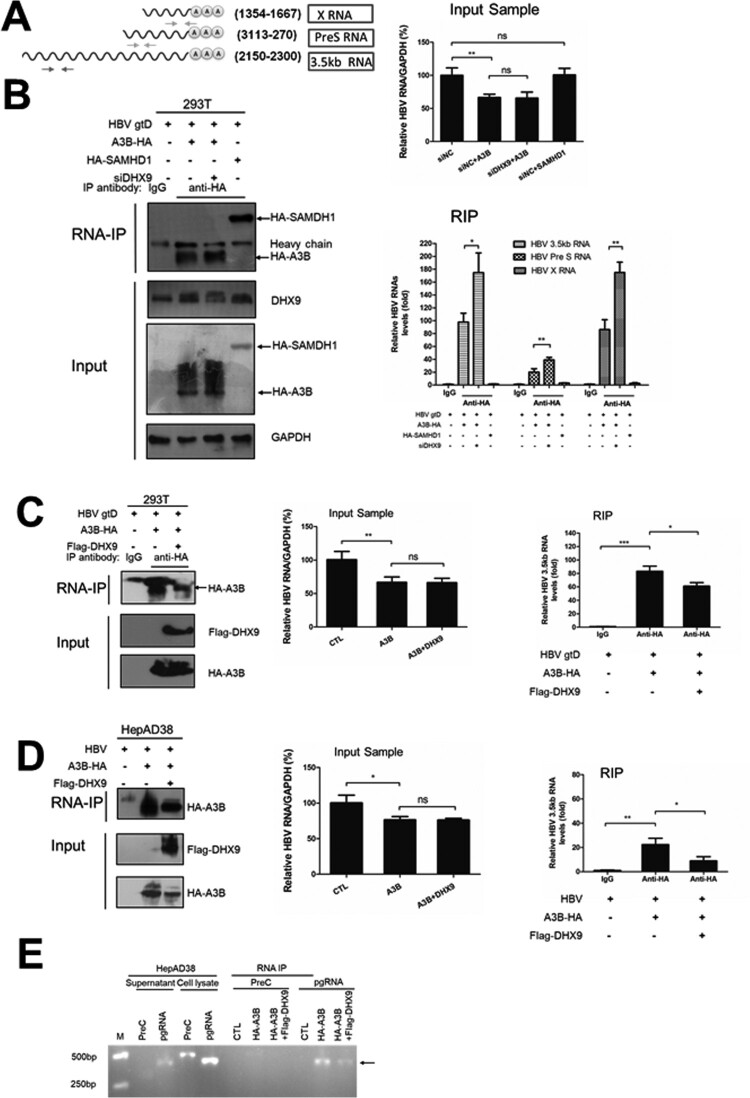
DHX9 inhibits the binding between A3B and HBV 3.5 kb RNA. (A) A diagram representing the primers used to quantify HBV mRNAs. (B) HEK293T cells were transfected with siRNA targeting DHX9 or control siRNA first and then cotransfected an HA-A3B or HA-SAMHD1 plasmid with HBV expression plasmid as indicated. Forty-eight hours later, the cell lysates were subjected to for RIP: aliquots (1/10 volume) of samples were detected by Western blotting for the desired proteins and in real-time RT-PCR for HBV RNA levels in the input sample, respectively. The remaining samples were incubated with magnetic beads for immunoprecipitation with an anti-HA antibody overnight. Aliquots (1/10 volume) of samples were detected by Western blotting and the remaining samples were subjected to proteinase K treatment and phenol/chloroform extraction. Normal mouse IgG was used as an isotype control. The relative levels of HBV RNA in the input samples or immunoprecipitated samples were assessed by real-time RT-PCR. (C) Overexpression of DHX9 reduced the binding levels of A3B and 3.5 kb RNA. HEK293T cells (C) or HepAD38 cells (D) were transfected with plasmids as indicated, and RIP was conducted as described in Figure 6(B). (E) Identification of A3B binding to HBV pgRNA but not preC mRNA. The HBV mRNA pulled down by anti-HA antibody in Figure 6(D) was examined by the primers described previously [14]. The cell lysate or the supernatants derived from HepAD38 cells were employed as a control. Statistical significance was determined by one-way ANOVA with Tukey’s post hoc test (*p < 0.05, **p < 0.01, ***p < 0.001, ns, not significant).

**Figure 7.**
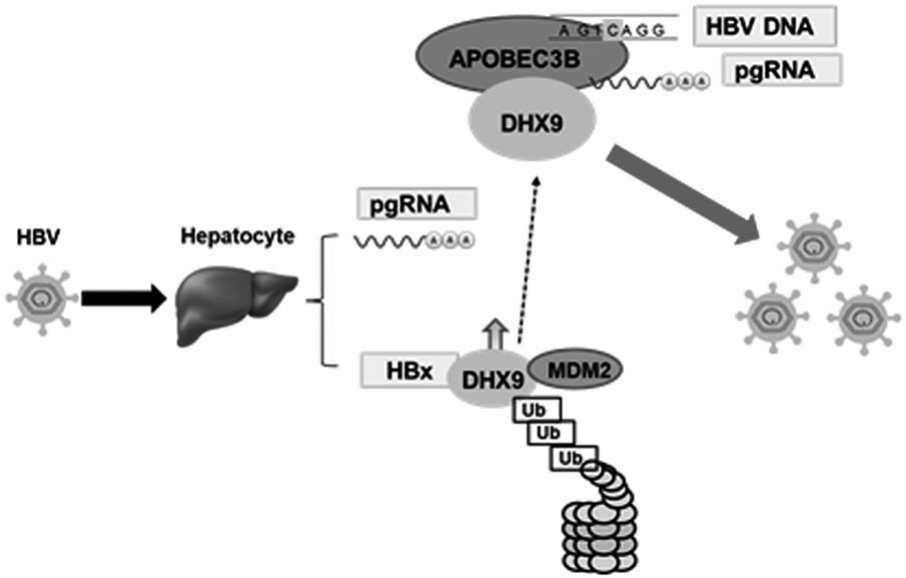
A model of the mechanism by which DHX9 attenuates the restriction of HBV replication by A3B. HBV infection induces upregulation of DHX9 by inhibiting its proteasome-dependent degradation mediated by MDM2, as demonstrated previously [20], then the upregulated DHX9 interacts with A3B and attenuates the binding of A3B with HBV pgRNA. As a consequence, DHX9 suppresses the anti-HBV function of A3B to promote viral replication.

Finally, we examined whether DHX9 affects the binding of A3B to viral RNAs. As HBV transcripts differ in their 5′ terminus but overlap at the 3′ terminus, we could use three sets of primers to amplify the three different HBV mRNAs (Figure 6(A)). To exclude nonspecific interactions of the anti-HA antibody with viral RNAs, HA-tagged SAMHD1, a host restriction factor of HBV, was also employed in the RNA-IP experiment. As shown in Figure 6(B), HA-A3B or HA-SAMHD1 could be pulled down by the anti-HA antibody (left panel); however, only the HBV mRNAs were enriched more than 20-fold in the HA-A3B precipitated group but not in HA-SAMHD1 group or control isotype antibody (right panel), even overexpression of A3B led to decreased HBV RNA levels in the input samples as measured by real-time RT-PCR. This indicated that HBV RNA transcripts were bound to A3B specifically. Moreover, the HBV mRNA transcripts precipitated with A3B could be amplified by both sets of primers, which indicated that the HBV mRNA transcripts bound to A3B should be HBV 3.5 kb RNA. Notably, silencing DHX9 increased the levels of HBV 3.5 kb RNA bound to A3B (right panel, Figure 6(B)). Conversely, ectopic expression of DHX9 suppressed this interaction of A3B and HBV 3.5 kb RNA (Figure 6(C)). Similar results were observed in HepAD38 cells (Figure 6(D)). These data suggest that DHX9 attenuates the binding between A3B and HBV 3.5 kb RNA. As HBV 3.5 kb RNA represents preC mRNA and pgRNA, and preC mRNA is longer than pgRNA by approximately 30 nucleotides, we further used RNA-specific primers to identify the HBV 3.5 kb RNA bound to A3B. As a control, only pgRNA, not preC mRNA was found in the supernatants of HepAD38 cells, which was in accordance with previous reports by Wang et al [14]. Using these primers, we determined that the HBV 3.5 kb RNA bound with A3B was pgRNA, not preC mRNA (Figure 6(E)). Overall, combined with the data in Figure 2, we conclude that during HBV infection, upregulated DHX9 binds to A3B and attenuates the binding of A3B and pgRNA, and as a consequence, DHX9 suppresses the anti-HBV effect of A3B to promote viral replication (Figure 7).

## Discussion

A3B is a key host restriction factor that has been demonstrated to remarkably inhibit HBV replication, which is dependent on its cytidine deaminase activity [13, 21]. A3B can be upregulated by LTβR agonists and is able to edit cccDNA in the nucleus [12], which provides a promising strategy to cure HBV [22]. However, its strong deaminase activity and predominantly nuclear location are potentially toxic and oncogenic to the host genome. Recently, several reports have demonstrated that A3B was upregulated in different tumours, such as breast cancer [18] and HBV-associated HCC [17], and this upregulation was correlated with genomic C to T mutations [18], implicating A3B is involved in in cancer mutagenesis and development [23]. In addition, the deaminase-independent role of A3B in contributing to HCC tumorigenesis and metastasis had also been demonstrated by Ma and colleagues [24]. Therefore, it is important to identify host factors that potentially interact with and regulate the function of A3B. Recently, hnRNP A3 and CDK4 have been suggested to interact with A3B [25, 26]; however, whether these factors affect the anti-HBV function of A3B is unclear. In this research, we employed a proteomic approach to identify and comprehensively compare host factors that potentially interact with A3B in the presence of HBV, and we identified eight distinct PPI networks, including mRNA metabolism, protein folding and viral process (Figure 1).

Among these factors, DHX9 was selected for further investigation, due to its most role in RNA metabolism including RNA processing [27] and the replication of viruses, such as HIV-1 [28], hepatitis C virus (HCV) [29] and Chikungunya virus [30]. Moreover, previously we demonstrated that the upregulation of DHX9 induced by HBx contributes to viral DNA replication [20]. Therefore, we extended this research to ask whether DHX9 contributes to viral DNA replication by interacting with A3B and attenuating the anti-HBV effect of A3B. First, we demonstrated that DHX9 can interact with A3B directly, and this binding was enhanced in the presence of HBV (Figure 2), confirming the LC-MS results. Next, the anti-HBV function of A3B was found to be enhanced by silencing the expression of DHX9. Conversely, ectopic expression of DHX9 suppressed the restriction by A3B of HBV replication, suggesting that DHX9 negatively regulates the anti-HBV function of A3B. Meanwhile, we just pointed out that DHX9 stimulated viral DNA replication partially by attenuating the anti-HBV efficacy of A3B, as in the A3B knockdown group, the contribution to viral DNA replication of DHX9 was not completely disappeared but partially reduced (Figure 3). Furthermore, we demonstrated that the interaction between A3B and DHX9 mostly relied on the RBD region in the N-terminus and RGG-box in the C-terminus of DHX9 (Figure 4). Deleting both of these regions abolished the antagonistic effect of DHX9 on the anti-HBV effect of A3B, suggesting that DHX9 attenuating the anti-HBV effect of A3B is dependent on their interaction. However, the binding of DHX9 to A3B had no effect on the deamination activity of A3B *in vitro* or the degree of viral DNA editing by A3B in the HBV infection model, as well as the interaction between the HBV core protein and A3B (Figure 5). Therefore, we asked whether DHX9 affects the association of viral RNAs with A3B. Using HBV-specific RNA primers to amplify the RNA-IP complex, we found that A3B is associated with HBV pgRNA. Although A3B does not edit HBV RNAs during reverse transcription, as demonstrated in our previous report [13], considering that A3B also edit cccDNA dependent on its deaminase activity in the nucleus [12], it is possible that A3B can access pgRNA during unwinding of the DNA strand in/after the process of cccDNA transcription. Meanwhile, DHX9 suppresses the binding of A3B with pgRNA without affecting deaminase activity of A3B, therefore, DHX9 had no potential effect on cccDNA levels or cccDNA transcription. This is consistent with our previous finding that DHX9 stimulates HBV DNA replication but not affect viral RNA levels [20]. Overall, combined with the fact that HBx induced upregulation of DHX9 through repressing its proteasome-dependent degradation by MDM2 [20], we propose a model in which during HBV infection with hepatocytes, as a host important restriction factor, A3B edits viral DNA to reduce viral DNA replication. In this process, the upregulated DHX9 could interact with A3B and inhibit the association between A3B/pgRNA and attenuate the anti-HBV efficacy of A3B, consequently contributing to viral DNA replication (Figure 7).

The function of the A3B protein is a double-edged sword, as it is characterized by powerful deaminase activity and predominantly nuclear localization; therefore, distinguishing the mechanism of host factors affecting the antiviral function of A3B either by regulating its cytidine deaminase activity or affecting the binding of A3B with core protein/viral RNAs would be helpful to design and develop new treatments to cure HBV infection. In this research, we employed PPI network analysis combined functional experiments to classify DHX9 as a negative regulator of the anti-HBV function of A3B in a manner independent of affecting the deaminase activity of A3B. Given that A3B potentially affects cancer development by editing genomic DNA and that DHX9 contributes to viral DNA replication [20], our comparative study uncovered new aspects of the potential function of DHX9 in regulating the anti-HBV function of A3B and would be helpful in the design strategies of targeting A3B to cure HBV infections.

## Supplementary Material

Supplemental Material
