# Supplementary material for: DHX9 interacts with APOBEC3B and attenuates the anti-HBV effect of APOBEC3B
Source: Emerg Microbes Infect. 2020 Feb 14;9(1):366–77. doi: 10.1080/22221751.2020.1725398 (PMC7033728; doi:10.1080/22221751.2020.1725398)
Supplement: Supplemental Material [file TEMI_A_1725398_SM3759.zip › Supplement_Table_1.doc]

**Supplement Table. 1 siRNA target sequence and the primers utilized in this study**

| Primer | Sequence (5’to 3’) |
| --- | --- |
| si-DHX9 FP | 5’-AAGAAGUGCAAGCGACUCUAGTT-3’ |
| si-DHX9 RP | 5’-CUAGAGUCGCUUGCACUUCUUTT-3’ |
| si-A3B FP | 5’-UGACCUACGAUGAGUUUGATT-3’ |
| si-A3B RP | 5’-UCAAACUCAUCGUAGGUCATT-3’ |
| HBV pgRNAFP | 5'-CCTAGTAGTCAGTTATGTCAAC -3' |
| HBV pgRNARP | 5'- TCTATAAGCTGGAGGAGTGCGA -3' |
| HBV XFP | 5’- CGCAAATATACATCGTATCCAT -3’ |
| HBV XRP | 5’- AAGAGTYYTYTTATGTAAGACYTT -3’ |
| HBV PreS1FP | 5'- TGGGGTGGAGCCCTCAVGCTCAGGGC -3' |
| HBV PreS1RP | 5'- AGAGAAGTCCACCACGAGTCTAGACTC -3' |
| DHX9FP | 5’-GGAAGCGAAGGCTGATCTGA-3’ |
| DHX9RP | 5’-TGGTGGAATTTCCCATGCCA-3’ |
| DHX9-△3-252FP | 5’- GGATGACGACGATAAGGGTGGAGGCGGTAGCGGTCTTGGAGTGGTTGAAGCTTACTCCGGACTTAC -3’ |
| DHX9-△3-252RP | 5’- GTAAGTCCGGAGTAAGCTTCAACCACTCCAAGACCGCTACCGCCTCCACCCTTATCGTCGTCATCC -3’ |
| DHX9-△1173-1260FP | 5’- GGCCCGATACGACAATGGAAGCGGATTTGGACAGGGAAGAGGAGGTGGCG -3’ |
| DHX9-△1173-1260RP | 5’- CGCCACCTCCTCTTCCCTGTCCAAATCCGCTTCCATTGTCGTATCGGGCC -3’ |
