# Supplementary material for: DHX9 interacts with APOBEC3B and attenuates the anti-HBV effect of APOBEC3B
Source: Emerg Microbes Infect. 2020 Feb 14;9(1):366–77. doi: 10.1080/22221751.2020.1725398 (PMC7033728; doi:10.1080/22221751.2020.1725398)
Supplement: Supplemental Material [file TEMI_A_1725398_SM3759.zip › Supplement_Table_2.doc]

**Supplement Table. 2 Proteins determined in mass spectrometric analysis of APOBEC3B-HA**

| A3B-interacting proteins in the absence of HBV by IP-MS | | | | | | | |
| --- | --- | --- | --- | --- | --- | --- | --- |
| Accession | Gene names | MW [kDa] | Protein score | Sequence coverage (%) | # Unique Peptides | # Peptides | # PSMs |
| P08670 | VIM | 53.62 | 967.02 | 44.42 | 21 | 21 | 26 |
| P0DMV9 | HSPA1B | 70.01 | 663.43 | 24.18 | 11 | 14 | 17 |
| P11940 | PABPC1 | 70.63 | 565.07 | 28.3 | 11 | 14 | 14 |
| P52272 | HNRNPM | 77.46 | 521.23 | 23.97 | 14 | 14 | 14 |
| P09651 | HNRNPA1 | 38.72 | 466.35 | 27.69 | 6 | 8 | 11 |
| Q12906 | ILF3 | 95.28 | 445.09 | 13.2 | 11 | 11 | 13 |
| P22626 | HNRNPA2B1 | 37.41 | 431.43 | 26.06 | 5 | 7 | 10 |
| Q9BQE3 | TUBA1C | 49.86 | 376.80 | 28.29 | 9 | 9 | 9 |
| P07910 | HNRNPC | 33.65 | 356.99 | 22.88 | 9 | 9 | 9 |
| P07437 | TUBB | 49.64 | 334.14 | 26.35 | 3 | 9 | 10 |
| Q12905 | ILF2 | 43.04 | 328.57 | 20 | 6 | 6 | 8 |
| P38159 | RBMX | 42.31 | 310.77 | 25.58 | 2 | 8 | 9 |
| P60709 | ACTB | 41.71 | 298.98 | 28 | 7 | 7 | 8 |
| P68371 | TUBB4B | 49.80 | 276.18 | 19.1 | 1 | 7 | 8 |
| O00571 | DDX3X | 73.20 | 276.15 | 12.08 | 7 | 7 | 7 |
| Q86YZ3 | HRNR | 282.23 | 268.39 | 9.89 | 4 | 4 | 5 |
| P67809 | YBX1 | 35.90 | 266.30 | 30.25 | 5 | 5 | 5 |
| P11142 | HSPA8 | 70.85 | 265.42 | 11.46 | 2 | 6 | 6 |
| P04406 | GAPDH | 36.03 | 258.68 | 20.3 | 6 | 6 | 6 |
| P60842 | EIF4A1 | 46.12 | 257.03 | 14.53 | 5 | 5 | 6 |
| P61978 | HNRNPK | 50.94 | 249.14 | 22.46 | 8 | 8 | 9 |
| P51991 | HNRNPA3 | 39.57 | 246.46 | 16.67 | 4 | 5 | 7 |
| P00338 | LDHA | 36.67 | 246.17 | 21.99 | 5 | 6 | 6 |
| P08238 | HSP90AB1 | 83.21 | 245.55 | 8.43 | 5 | 6 | 6 |
| P36578 | RPL4 | 47.67 | 235.44 | 17.1 | 7 | 7 | 7 |
| P07195 | LDHB | 36.62 | 228.61 | 16.17 | 3 | 4 | 4 |
| Q96E39 | RBMXL1 | 42.12 | 227.17 | 22.31 | 1 | 7 | 7 |
| P11021 | HSPA5 | 72.29 | 209.60 | 8.1 | 2 | 4 | 4 |
| P38646 | HSPA9 | 73.63 | 193.39 | 6.19 | 3 | 3 | 3 |
| Q13310 | PABPC4 | 70.74 | 190.37 | 8.23 | 1 | 4 | 4 |
| Q99623 | PHB2 | 33.28 | 190.25 | 15.05 | 4 | 4 | 4 |
| P68104 | EEF1A1 | 50.11 | 189.82 | 10.39 | 3 | 3 | 5 |
| Q00839 | HNRNPU | 90.53 | 179.10 | 5.21 | 3 | 3 | 3 |
| P31943 | HNRNPH1 | 49.20 | 174.79 | 10.24 | 3 | 3 | 3 |
| P14618 | PKM | 57.90 | 171.54 | 9.04 | 4 | 4 | 4 |
| P06576 | ATP5B | 56.52 | 164.43 | 9.07 | 4 | 4 | 4 |
| P62701 | RPS4X | 29.58 | 163.95 | 15.97 | 5 | 5 | 5 |
| O43390 | HNRNPR | 70.90 | 154.33 | 8.85 | 4 | 4 | 4 |
| Q02878 | RPL6 | 32.71 | 136.71 | 14.24 | 3 | 3 | 3 |
| P62826 | RAN | 24.41 | 132.55 | 16.2 | 3 | 3 | 3 |
| P62753 | RPS6 | 28.66 | 131.80 | 13.25 | 3 | 3 | 3 |
| P18124 | RPL7 | 29.21 | 125.97 | 11.69 | 3 | 3 | 3 |
| P61313 | RPL15 | 24.13 | 125.34 | 11.27 | 2 | 2 | 2 |
| P19338 | NCL | 76.57 | 123.43 | 4.23 | 3 | 3 | 3 |
| P26373 | RPL13 | 24.25 | 121.92 | 14.69 | 3 | 3 | 3 |
| P16403 | HIST1H1C | 21.35 | 118.70 | 15.02 | 1 | 3 | 3 |
| P10412 | HIST1H1E | 21.85 | 116.80 | 14.61 | 1 | 3 | 3 |
| Q9NZI8 | IGF2BP1 | 63.44 | 116.04 | 4.68 | 2 | 2 | 2 |
| Q13151 | HNRNPA0 | 30.82 | 115.49 | 6.56 | 1 | 2 | 4 |
| P50914 | RPL14 | 23.42 | 113.54 | 16.28 | 3 | 3 | 3 |
| Q9UKM9 | RALY | 32.44 | 110.78 | 9.15 | 3 | 3 | 3 |
| O14979 | HNRNPDL | 46.41 | 108.79 | 6.19 | 2 | 2 | 2 |
| P52597 | HNRNPF | 45.64 | 106.29 | 3.86 | 1 | 1 | 2 |
| P62195 | PSMC5 | 45.60 | 102.85 | 3.2 | 1 | 1 | 1 |
| P25705 | ATP5A1 | 59.71 | 100.50 | 3.25 | 2 | 2 | 2 |
| Q92841 | DDX17 | 80.22 | 100.10 | 3.29 | 2 | 2 | 2 |
| P31942 | HNRNPH3 | 36.90 | 99.69 | 4.05 | 1 | 1 | 1 |
| P14625 | HSP90B1 | 92.41 | 98.05 | 2.86 | 1 | 2 | 2 |
| P62241 | RPS8 | 24.19 | 97.80 | 12.02 | 2 | 2 | 2 |
| P84098 | RPL19 | 23.45 | 96.38 | 8.67 | 1 | 1 | 1 |
| P35232 | PHB | 29.79 | 95.86 | 8.09 | 2 | 2 | 2 |
| P05141 | SLC25A5 | 32.83 | 93.75 | 8.39 | 2 | 2 | 2 |
| Q15233 | NONO | 54.20 | 93.15 | 5.1 | 2 | 2 | 2 |
| Q96KR1 | ZFR | 116.94 | 89.54 | 1.58 | 1 | 1 | 1 |
| Q07021 | C1QBP | 31.34 | 89.14 | 8.51 | 2 | 2 | 2 |
| P26641 | EEF1G | 50.09 | 89.03 | 5.26 | 2 | 2 | 2 |
| P81605 | DCD | 11.28 | 83.07 | 20 | 2 | 2 | 2 |
| Q5D862 | FLG2 | 247.93 | 82.87 | 0.96 | 2 | 2 | 2 |
| P62917 | RPL8 | 28.01 | 80.76 | 8.95 | 2 | 2 | 2 |
| A0A075B6S6 | IGKV2D-30 | 13.21 | 80.49 | 10.83 | 1 | 1 | 1 |
| P49368 | CCT3 | 60.50 | 78.77 | 4.04 | 2 | 2 | 2 |
| Q9P035 | HACD3 | 43.13 | 78.04 | 3.87 | 1 | 1 | 1 |
| P15880 | RPS2 | 31.30 | 77.89 | 6.83 | 2 | 2 | 2 |
| P10809 | HSPD1 | 61.02 | 77.47 | 5.24 | 2 | 2 | 2 |
| P06733 | ENO1 | 47.14 | 75.43 | 3.92 | 2 | 2 | 2 |
| Q96PK6 | RBM14 | 69.45 | 73.56 | 3.59 | 2 | 2 | 2 |
| P63244 | RACK1 | 35.05 | 69.85 | 5.99 | 2 | 2 | 2 |
| Q92522 | H1FX | 22.47 | 68.65 | 4.69 | 1 | 1 | 1 |
| Q92804 | TAF15 | 61.79 | 68.47 | 6.08 | 1 | 1 | 1 |
| P27348 | YWHAQ | 27.75 | 68.06 | 2.86 | 1 | 1 | 1 |
| Q9NR30 | DDX21 | 87.29 | 67.70 | 2.68 | 2 | 2 | 2 |
| P51148 | RAB5C | 23.47 | 67.30 | 5.56 | 1 | 1 | 1 |
| P27635 | RPL10 | 24.59 | 66.59 | 6.07 | 1 | 1 | 1 |
| P62424 | RPL7A | 29.98 | 63.95 | 9.4 | 2 | 2 | 2 |
| O43143 | DHX15 | 90.88 | 62.29 | 1.51 | 1 | 1 | 1 |
| P46777 | RPL5 | 34.34 | 59.49 | 4.04 | 1 | 1 | 1 |
| P14678 | SNRPB | 24.59 | 57.77 | 3.33 | 1 | 1 | 1 |
| Q07020 | RPL18 | 21.62 | 55.93 | 5.85 | 1 | 1 | 1 |
| A6NMY6 | ANXA2P2 | 38.63 | 55.48 | 2.95 | 1 | 1 | 1 |
| P0CG48 | UBC | 76.99 | 55.28 | 11.82 | 1 | 1 | 1 |
| P05388 | RPLP0 | 34.25 | 55.10 | 3.79 | 1 | 1 | 1 |
| P38919 | EIF4A3 | 46.84 | 54.25 | 3.16 | 1 | 1 | 1 |
| Q96HS1 | PGAM5 | 31.98 | 53.51 | 4.15 | 1 | 1 | 1 |
| P22087 | FBL | 33.76 | 52.78 | 3.43 | 1 | 1 | 1 |
| O14980 | XPO1 | 123.31 | 52.71 | 0.65 | 1 | 1 | 1 |
| O00148 | DDX39A | 49.10 | 52.20 | 2.34 | 1 | 1 | 1 |
| P17987 | TCP1 | 60.31 | 51.88 | 1.44 | 1 | 1 | 1 |
| P02748 | C9 | 63.13 | 51.66 | 1.61 | 1 | 1 | 1 |
| P23396 | RPS3 | 26.67 | 51.64 | 3.7 | 1 | 1 | 1 |
| P04792 | HSPB1 | 22.77 | 50.54 | 4.88 | 1 | 1 | 1 |
| P30041 | PRDX6 | 25.02 | 47.45 | 4.02 | 1 | 1 | 1 |
| P50991 | CCT4 | 57.89 | 47.13 | 1.48 | 1 | 1 | 1 |
| P06748 | NPM1 | 32.55 | 47.04 | 2.38 | 1 | 1 | 1 |
| P02768 | ALB | 69.32 | 46.76 | 2.46 | 1 | 1 | 1 |
| Q5T2N8 | ATAD3C | 46.35 | 46.26 | 1.95 | 1 | 1 | 1 |
| P14923 | JUP | 81.69 | 45.63 | 0.94 | 1 | 1 | 1 |
| Q9NTU7 | CBLN4 | 21.79 | 45.05 | 3.48 | 1 | 1 | 1 |
| P23526 | AHCY | 47.69 | 43.43 | 1.85 | 1 | 1 | 1 |
| P15924 | DSP | 331.57 | 43.03 | 0.42 | 1 | 1 | 1 |
| Q13242 | SRSF9 | 25.53 | 42.68 | 4.07 | 1 | 1 | 1 |
| P39748 | FEN1 | 42.57 | 40.80 | 2.89 | 1 | 1 | 1 |
| Q96DV4 | MRPL38 | 44.57 | 39.84 | 1.84 | 1 | 1 | 1 |
| P54886 | ALDH18A1 | 87.25 | 35.02 | 0.88 | 1 | 1 | 1 |

| A3B-interacting proteins in the presence of HBV by IP-MS | | | | | | | |
| --- | --- | --- | --- | --- | --- | --- | --- |
| Accession | Gene names | Score | Coverage | # Unique Peptides | # Peptides | # PSMs | MW [kDa] |
| P38646 | GRP75 | 2291.70 | 35.05 | 22 | 22 | 51 | 73.63 |
| P0DMV9 | HSPA1B | 1045.17 | 27.15 | 12 | 15 | 26 | 70.01 |
| P11142 | HSPA8 | 959.58 | 28.17 | 13 | 17 | 24 | 70.85 |
| P11021 | HSPA5 | 763.33 | 30.12 | 14 | 16 | 19 | 72.29 |
| Q00839 | HNRNPU | 591.83 | 13.45 | 10 | 10 | 13 | 90.53 |
| P35637 | FUS | 575.27 | 12.55 | 6 | 8 | 13 | 53.39 |
| Q08211 | DHX9 | 543.80 | 10.39 | 11 | 11 | 13 | 140.87 |
| P08238 | HSP90AB1 | 509.47 | 20.44 | 5 | 13 | 13 | 83.21 |
| Q12906 | ILF3 | 411.99 | 11.41 | 10 | 10 | 11 | 95.28 |
| O00571 | DDX3X | 403.47 | 17.82 | 11 | 11 | 11 | 73.20 |
| P07900 | HSP90AA1 | 395.61 | 14.07 | 2 | 9 | 9 | 84.61 |
| P21333 | FLNA | 366.07 | 5.18 | 9 | 9 | 9 | 280.56 |
| Q9BUJ2 | HNRNPUL1 | 356.05 | 10.51 | 7 | 7 | 9 | 95.68 |
| Q86YZ3 | HRNR | 338.62 | 6.63 | 4 | 4 | 7 | 282.23 |
| P11940 | PABPC1 | 334.21 | 11.01 | 6 | 7 | 8 | 70.63 |
| Q01844 | EWSR1 | 330.66 | 10.37 | 5 | 5 | 6 | 68.44 |
| Q92734 | TFG | 323.55 | 18.75 | 5 | 5 | 7 | 43.42 |
| Q9NZI8 | IGF2BP1 | 291.48 | 14.38 | 6 | 8 | 9 | 63.44 |
| P61978 | HNRNPK | 278.87 | 15.55 | 6 | 6 | 7 | 50.94 |
| P14625 | HSP90B1 | 274.73 | 9.71 | 6 | 7 | 7 | 92.41 |
| P68371 | TUBB4B | 267.67 | 12.58 | 1 | 5 | 7 | 49.80 |
| P07437 | TUBB | 265.60 | 12.61 | 1 | 5 | 7 | 49.64 |
| P19338 | NCL | 264.88 | 10.56 | 7 | 7 | 7 | 76.57 |
| P08621 | SNRNP70 | 217.92 | 9.84 | 4 | 4 | 5 | 51.53 |
| P49327 | FASN | 215.61 | 2.67 | 6 | 6 | 6 | 273.25 |
| Q9BQE3 | TUBA1C | 200.27 | 11.8 | 4 | 4 | 4 | 49.86 |
| P16989 | YBX3 | 198.98 | 15.32 | 4 | 4 | 4 | 40.07 |
| P02768 | ALB | 188.42 | 3.61 | 2 | 2 | 4 | 69.32 |
| P13639 | EEF2 | 187.31 | 6.29 | 5 | 5 | 5 | 95.28 |
| O43175 | PHGDH | 181.91 | 7.32 | 3 | 3 | 3 | 56.61 |
| P56945 | BCAR1 | 180.37 | 5.98 | 3 | 3 | 3 | 93.31 |
| O60506 | SYNCRIP | 179.50 | 5.3 | 2 | 3 | 5 | 69.56 |
| P50991 | CCT4 | 173.41 | 7.98 | 4 | 4 | 4 | 57.89 |
| P17844 | DDX5 | 151.87 | 6.84 | 3 | 4 | 4 | 69.10 |
| O43390 | HNRNPR | 151.07 | 5.21 | 2 | 3 | 4 | 70.90 |
| Q13310 | PABPC4 | 147.93 | 5.75 | 2 | 3 | 3 | 70.74 |
| P14618 | PKM | 145.24 | 8.29 | 3 | 3 | 3 | 57.90 |
| Q00610 | CLTC | 141.51 | 2.33 | 3 | 3 | 3 | 191.49 |
| Q8NC51 | SERBP1 | 140.12 | 6.62 | 2 | 2 | 2 | 44.94 |
| P17987 | TCP1 | 139.36 | 5.94 | 3 | 3 | 3 | 60.31 |
| P53992 | SEC24C | 139.01 | 3.66 | 3 | 3 | 3 | 118.25 |
| O00425 | IGF2BP3 | 137.12 | 5.7 | 1 | 3 | 4 | 63.67 |
| Q12931 | TRAP1 | 129.98 | 3.84 | 1 | 2 | 2 | 80.06 |
| Q92841 | DDX17 | 127.07 | 4.25 | 2 | 3 | 3 | 80.22 |
| Q99613 | EIF3C | 126.61 | 3.72 | 3 | 3 | 3 | 105.28 |
| O75533 | SF3B1 | 126.43 | 1.46 | 1 | 1 | 2 | 145.74 |
| Q92804 | TAF15 | 124.72 | 6.93 | 1 | 3 | 3 | 61.79 |
| Q13263 | TRIM28 | 116.41 | 3.47 | 2 | 2 | 2 | 88.49 |
| P55786 | NPEPPS | 114.25 | 2.72 | 2 | 2 | 2 | 103.21 |
| P50990 | CCT8 | 111.80 | 5.29 | 2 | 2 | 2 | 59.58 |
| Q53GT1 | KLHL22 | 111.29 | 4.73 | 2 | 2 | 2 | 71.62 |
| P58107 | EPPK1 | 109.92 | 1.63 | 2 | 2 | 2 | 555.28 |
| Q6P2Q9 | PRPF8 | 103.57 | 0.73 | 2 | 2 | 2 | 273.43 |
| Q9HCE1 | MOV10 | 101.83 | 2.89 | 2 | 2 | 2 | 113.60 |
| P13797 | PLS3 | 100.08 | 4.13 | 2 | 2 | 2 | 70.77 |
| Q9UKV8 | AGO2 | 97.87 | 3.03 | 2 | 2 | 2 | 97.15 |
| Q8WU90 | ZC3H15 | 97.75 | 3.29 | 1 | 1 | 1 | 48.57 |
| Q9NTJ3 | SMC4 | 96.18 | 1.55 | 2 | 2 | 2 | 147.09 |
| P31943 | HNRNPH1 | 95.97 | 3.56 | 1 | 1 | 1 | 49.20 |
| P35269 | GTF2F1 | 94.31 | 3.09 | 1 | 1 | 1 | 58.21 |
| Q13435 | SF3B2 | 94.13 | 2.35 | 2 | 2 | 2 | 100.16 |
| P25205 | MCM3 | 93.87 | 3.09 | 2 | 2 | 2 | 90.92 |
| P40227 | CCT6A | 90.99 | 3.95 | 2 | 2 | 2 | 57.99 |
| P04843 | RPN1 | 89.51 | 3.79 | 2 | 2 | 2 | 68.53 |
| Q08380 | LGALS3BP | 89.45 | 4.27 | 2 | 2 | 2 | 65.29 |
| Q8IXB1 | DNAJC10 | 86.87 | 2.52 | 2 | 2 | 2 | 91.02 |
| Q86X55 | CARM1 | 84.16 | 3.13 | 2 | 2 | 2 | 65.81 |
| P09874 | PARP1 | 83.45 | 2.76 | 2 | 2 | 2 | 113.01 |
| Q9UHX1 | PUF60 | 83.16 | 4.83 | 2 | 2 | 2 | 59.84 |
| P14866 | HNRNPL | 82.53 | 3.06 | 2 | 2 | 2 | 64.09 |
| P35579 | MYH9 | 82.26 | 1.02 | 2 | 2 | 2 | 226.39 |
| P46940 | IQGAP1 | 81.35 | 1.09 | 2 | 2 | 2 | 189.13 |
| Q92945 | KHSRP | 81.20 | 2.81 | 2 | 2 | 2 | 73.07 |
| P52272 | HNRNPM | 80.36 | 2.74 | 2 | 2 | 2 | 77.46 |
| P78527 | PRKDC | 79.69 | 0.44 | 2 | 2 | 2 | 468.79 |
| P35573 | AGL | 79.59 | 1.24 | 2 | 2 | 2 | 174.65 |
| Q15233 | NONO | 79.29 | 3.61 | 1 | 2 | 2 | 54.20 |
| P55072 | VCP | 79.15 | 3.1 | 2 | 2 | 2 | 89.27 |
| P28799 | GRN | 78.61 | 3.88 | 2 | 2 | 2 | 63.50 |
| P49368 | CCT3 | 77.72 | 2.02 | 1 | 1 | 1 | 60.50 |
| P23246 | SFPQ | 74.56 | 2.26 | 1 | 2 | 2 | 76.10 |
| P01023 | A2M | 73.49 | 1.42 | 2 | 2 | 2 | 163.19 |
| P15924 | DSP | 73.00 | 0.56 | 2 | 2 | 2 | 331.57 |
| Q02413 | DSG1 | 72.03 | 1.53 | 1 | 1 | 1 | 113.68 |
| Q07666 | KHDRBS1 | 71.21 | 5.42 | 2 | 2 | 2 | 48.20 |
| Q01581 | HMGCS1 | 70.53 | 2.31 | 1 | 1 | 1 | 57.26 |
| P48643 | CCT5 | 68.63 | 1.85 | 1 | 1 | 1 | 59.63 |
| Q07065 | CKAP4 | 68.02 | 1.5 | 1 | 1 | 1 | 65.98 |
| O43896 | KIF1C | 67.88 | 1 | 1 | 1 | 1 | 122.87 |
| Q14247 | CTTN | 67.33 | 2.55 | 1 | 2 | 2 | 61.55 |
| Q14157 | UBAP2L | 65.39 | 1.38 | 1 | 1 | 1 | 114.47 |
| Q13283 | G3BP1 | 63.27 | 2.58 | 1 | 1 | 1 | 52.13 |
| P07384 | CAPN1 | 63.18 | 1.4 | 1 | 1 | 1 | 81.84 |
| Q9NZB2 | FAM120A | 62.84 | 1.16 | 1 | 1 | 1 | 121.81 |
| Q14697 | GANAB | 62.66 | 0.74 | 1 | 1 | 1 | 106.81 |
| P07237 | P4HB | 60.47 | 2.36 | 1 | 1 | 1 | 57.08 |
| P16615 | ATP2A2 | 59.95 | 1.25 | 1 | 1 | 1 | 114.68 |
| Q15436 | SEC23A | 59.72 | 1.44 | 1 | 1 | 1 | 86.11 |
| P53621 | COPA | 58.99 | 0.98 | 1 | 1 | 1 | 138.26 |
| Q96QK1 | VPS35 | 58.79 | 1.88 | 1 | 1 | 1 | 91.65 |
| Q9Y3I0 | RTCB | 58.61 | 1.78 | 1 | 1 | 1 | 55.17 |
| P26599 | PTBP1 | 58.04 | 2.07 | 1 | 1 | 1 | 57.19 |
| P68104 | EEF1A1 | 57.85 | 2.38 | 1 | 1 | 1 | 50.11 |
| P33993 | MCM7 | 57.41 | 1.81 | 1 | 1 | 1 | 81.26 |
| Q8N1G4 | LRRC47 | 56.31 | 2.06 | 1 | 1 | 1 | 63.43 |
| P31948 | STIP1 | 56.26 | 1.66 | 1 | 1 | 1 | 62.60 |
| P41252 | IARS | 56.11 | 0.79 | 1 | 1 | 1 | 144.41 |
| Q09028 | RBBP4 | 55.82 | 3.29 | 1 | 1 | 1 | 47.63 |
| Q7KZF4 | SND1 | 55.46 | 1.32 | 1 | 1 | 1 | 101.93 |
| P13667 | PDIA4 | 54.96 | 1.86 | 1 | 1 | 1 | 72.89 |
| Q9Y285 | FARSA | 54.89 | 2.17 | 1 | 1 | 1 | 57.53 |
| Q04637 | EIF4G1 | 54.24 | 0.56 | 1 | 1 | 1 | 175.38 |
| Q86VP6 | CAND1 | 53.64 | 1.22 | 1 | 1 | 1 | 136.29 |
| P09884 | POLA1 | 53.62 | 0.48 | 1 | 1 | 1 | 165.81 |
| Q16630 | CPSF6 | 53.55 | 2.54 | 1 | 1 | 1 | 59.17 |
| Q8WXF1 | PSPC1 | 53.24 | 2.1 | 1 | 1 | 1 | 58.71 |
| P30419 | NMT1 | 53.21 | 2.62 | 1 | 1 | 1 | 56.77 |
| Q14444 | CAPRIN1 | 52.95 | 1.69 | 1 | 1 | 1 | 78.32 |
| P22314 | UBA1 | 52.66 | 1.13 | 1 | 1 | 1 | 117.77 |
| P11586 | MTHFD1 | 52.65 | 1.28 | 1 | 1 | 1 | 101.50 |
| P07814 | EPRS | 52.53 | 0.6 | 1 | 1 | 1 | 170.48 |
| Q1KMD3 | HNRNPUL2 | 52.31 | 1.2 | 1 | 1 | 1 | 85.05 |
| P05023 | ATP1A1 | 52.22 | 0.98 | 1 | 1 | 1 | 112.82 |
| P42704 | LRPPRC | 52.13 | 0.65 | 1 | 1 | 1 | 157.81 |
| P47897 | QARS | 52.07 | 1.16 | 1 | 1 | 1 | 87.74 |
| P25705 | ATP5F1A | 52.00 | 1.81 | 1 | 1 | 1 | 59.71 |
| P26639 | TARS | 51.98 | 1.24 | 1 | 1 | 1 | 83.38 |
| Q08J23 | NSUN2 | 51.97 | 1.43 | 1 | 1 | 1 | 86.42 |
| O76031 | CLPX | 51.43 | 2.37 | 1 | 1 | 1 | 69.18 |
| P78371 | CCT2 | 51.25 | 2.06 | 1 | 1 | 1 | 57.45 |
| Q96P20 | NLRP3 | 51.20 | 1.06 | 1 | 1 | 1 | 118.10 |
| O75534 | CSDE1 | 50.86 | 1 | 1 | 1 | 1 | 88.83 |
| Q13200 | PSMD2 | 50.61 | 0.99 | 1 | 1 | 1 | 100.14 |
| P26038 | MSN | 50.22 | 1.73 | 1 | 1 | 1 | 67.78 |
| Q14566 | MCM6 | 50.11 | 1.58 | 1 | 1 | 1 | 92.83 |
| Q99459 | CDC5L | 50.07 | 1.25 | 1 | 1 | 1 | 92.19 |
| P81605 | DCD | 49.38 | 10 | 1 | 1 | 1 | 11.28 |
| Q7L2E3 | DHX30 | 49.35 | 0.67 | 1 | 1 | 1 | 133.85 |
| O43242 | PSMD3 | 48.81 | 2.43 | 1 | 1 | 1 | 60.94 |
| P34932 | HSPA4 | 48.42 | 1.55 | 1 | 1 | 1 | 94.27 |
| O15067 | PFAS | 48.06 | 0.82 | 1 | 1 | 1 | 144.64 |
| P46060 | RANGAP1 | 47.97 | 2.04 | 1 | 1 | 1 | 63.50 |
| Q6PKG0 | LARP1 | 47.87 | 1.19 | 1 | 1 | 1 | 123.43 |
| Q92499 | DDX1 | 47.67 | 1.49 | 1 | 1 | 1 | 82.38 |
| P27708 | CAD | 47.53 | 0.54 | 1 | 1 | 1 | 242.83 |
| Q9BYJ9 | YTHDF1 | 46.90 | 2.33 | 1 | 1 | 1 | 60.84 |
| Q9UHB9 | SRP68 | 46.71 | 1.44 | 1 | 1 | 1 | 70.69 |
| P12268 | IMPDH2 | 46.45 | 2.14 | 1 | 1 | 1 | 55.77 |
| B9A064 | IGLL5 | 45.96 | 3.74 | 1 | 1 | 1 | 23.05 |
| Q14204 | DYNC1H1 | 45.91 | 0.17 | 1 | 1 | 1 | 532.07 |
| P55060 | CSE1L | 45.46 | 0.82 | 1 | 1 | 1 | 110.35 |
| P16403 | HIST1H1C | 45.21 | 4.69 | 1 | 1 | 1 | 21.35 |
| O95163 | ELP1 | 42.43 | 0.53 | 1 | 1 | 1 | 150.16 |
| P10809 | HSPD1 | 41.44 | 1.22 | 1 | 1 | 1 | 61.02 |
| Q02790 | FKBP4 | 41.33 | 4.14 | 1 | 1 | 1 | 51.77 |
| Q8TCJ2 | STT3B | 40.92 | 1.45 | 1 | 1 | 1 | 93.61 |
| P52701 | MSH6 | 38.56 | 0.44 | 1 | 1 | 1 | 152.69 |
| Q9Y4Z0 | LSM4 | 38.08 | 5.04 | 1 | 1 | 1 | 15.34 |
| Q9P2J5 | LARS | 33.16 | 0.6 | 1 | 1 | 1 | 134.38 |
